# *Achyranthes bidentata*–functionalized bone organoids enhance bone defect repair

**DOI:** 10.1016/j.mtbio.2026.103716

**Published:** 2026-09-28

**Authors:** Chenxing Lu, Xudong Qin, Zihao Lin, Song Han, Peng Ding, Li Xu, Xu Zheng, Bin Hu, Fenghua Chen, Di Wu

**Affiliations:** a Department of Orthopedic Surgery, Taizhou Hospital of Traditional Chinese Medicine, Jiangsu, China; b School of Pharmaceutical Sciences, Guangzhou Medical University, Guangzhou, China; c Department of Orthopaedic Surgery, The First Affiliated Hospital of Ningbo University, Ningbo, Zhejiang, China; d Department of Orthopedics, Renji Hospital, Shanghai Jiao Tong University School of Medicine, Shanghai, China; e Institute of Microsurgery on Extremities, Shanghai Sixth People's Hospital Affiliated to Shanghai Jiao Tong University School of Medicine, Shanghai, China; f Department of Orthopedic Surgery, Shanghai Sixth People's Hospital Affiliated to Shanghai Jiao Tong University School of Medicine, Shanghai, China; g Division of Engineering in Medicine, Department of Medicine, Brigham and Women's Hospital, Harvard Medical School, Cambridge, MA, USA

**Keywords:** Bone organoids, *Achyranthes bidentata*, Bone defect repair, Osteogenic differentiation, Fracture healing

## Abstract

Large bone defects remain difficult to reconstruct because impaired osteogenesis, dysregulated remodeling, and an unfavorable local microenvironment jointly limit durable healing. Here, we developed a translational strategy that integrates *Achyranthes bidentata* (AB), a traditional osteoactive botanical factor, into a BMSC-derived bone organoid platform to enhance bone regeneration. We first screened AB across a concentration gradient and identified 200 μg/mL as the selected working concentration for promoting BMSC viability, osteogenic differentiation, and matrix mineralization, whereas higher concentrations showed diminished benefit. We then incorporated AB into three-dimensional bone organoid culture to generate AB-bone organoids, which exhibited enhanced viability, increased alkaline phosphatase (ALP) activity and Alizarin Red S (ARS) staining, greater COL-I deposition, and stronger osteogenic marker expression than untreated bone organoids. Functionally, AB-bone organoids outperformed both conventional monolayer BMSCs and untreated organoids, indicating that AB establishes a more potent osteogenic niche within the organoid microenvironment. In a critical-sized calvarial defect model, implantation of AB-bone organoids significantly improved new bone formation, defect bridging, collagen matrix deposition, and osteogenic marker expression compared with untreated organoids or empty defects. As an exploratory clinical extension, we analyzed a retrospective postoperative tibial-fracture cohort and found that systemic oral AB administration was associated with shorter radiographic healing time, higher mRUST scores, lower pain, and better lower-extremity function. Together, this study identifies AB as an organoid-compatible osteoregenerative cue that functionally upgrades bone organoids and supports the translational potential of AB-based organoid engineering for bone repair.

## Graphical abstract

**Figure:**
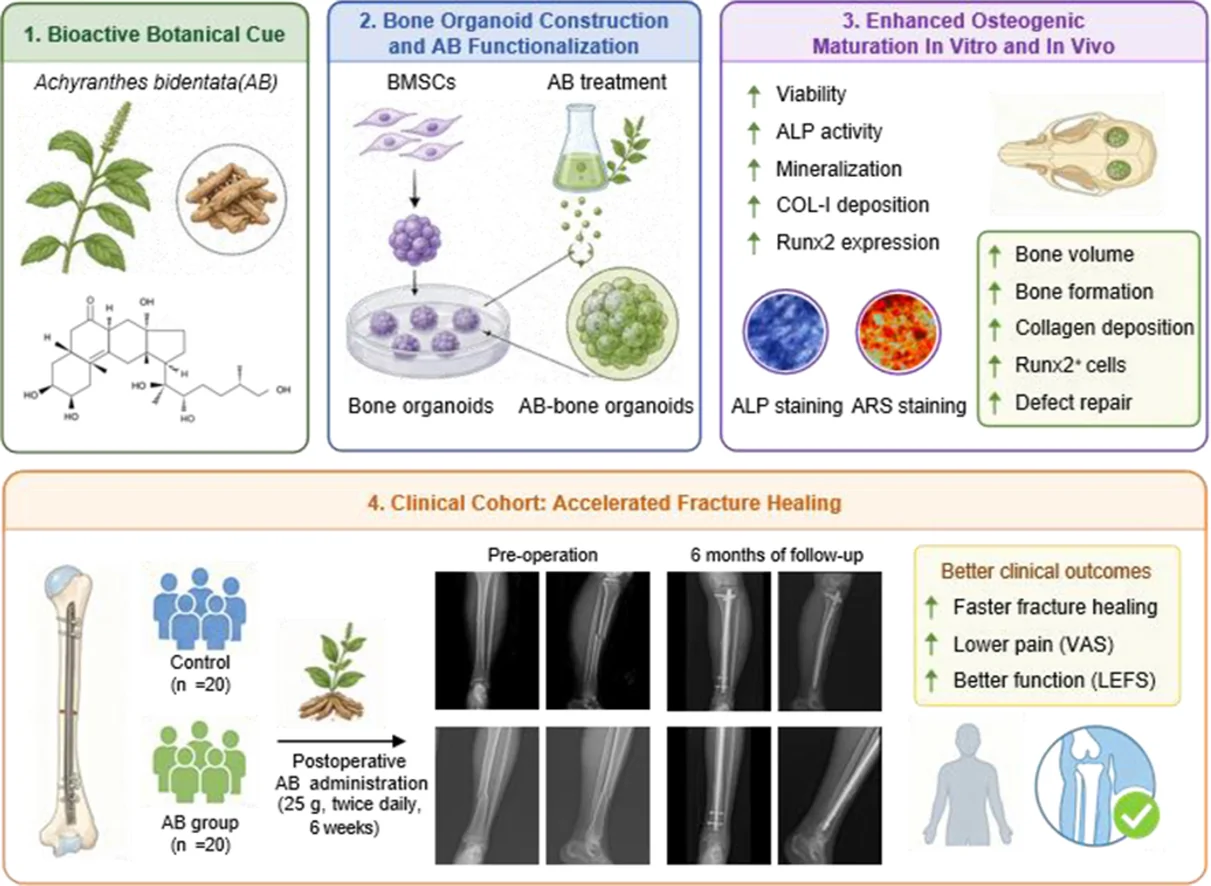

## 1 Introduction

Large bone defects remain a major problem in orthopaedic reconstruction. Trauma, tumor resection, infection, and repeated debridement can remove bone beyond the threshold of spontaneous healing, resulting in prolonged disability, repeated operations, and unsatisfactory structural restoration [1,2]. Current reconstructive options are still limited. Autologous bone grafting is constrained by donor-site morbidity and restricted tissue availability, whereas allografts carry risks of immune incompatibility, incomplete incorporation, and disease transmission [3,4]. Osteoinductive biologics such as bone morphogenetic proteins have expanded the therapeutic repertoire, yet their efficacy often depends on high local doses and may be accompanied by inflammation, ectopic ossification, and substantial cost. These limitations underscore the need for regenerative strategies that can simultaneously restore tissue architecture, support osteogenic maturation, and remain clinically scalable [5,6].

Organoid technology has created a new framework for musculoskeletal regeneration by moving beyond flat cell culture toward self-organized three-dimensional microtissues that better reproduce native tissue structure and function. In contrast to conventional two-dimensional culture, bone organoids preserve cell-cell communication, cell-matrix interactions, lineage patterning, and extracellular matrix remodeling within a spatially relevant environment [7,8]. Recent advances have broadened skeletal organoid systems to include bone marrow-like, trabecular-like, callus-like, and woven-bone-like constructs, while matrix engineering and biofabrication have enabled tighter control of organoid geometry and maturation [9,10]. However, the field still faces key barriers, particularly inadequate vascularization, immature mechanics, diffusion limitations in larger constructs, and the lack of standardized bioactive programming. In the present study, bone organoids refer to BMSC-derived three-dimensional osteogenic microtissues that reproduce selected features of bone formation, including cell-cell interaction, extracellular matrix production, and mineral deposition, rather than fully vascularized or marrow-containing bone equivalents.

*Achyranthes bidentata* (AB), the botanical source of the traditional Chinese medicine, is an attractive candidate for such biofunctionalization. Pharmacognostic studies identify multiple bioactive constituents in AB, including polysaccharides, saponins, steroids, and related small molecules, and toxicology work suggests a favorable safety profile at therapeutic doses [11,12]. Experimental studies further support a direct role in skeletal repair. AB saponins promote osteogenic differentiation of bone marrow stromal cells through the ERK/MAPK pathway, while osteoblast studies show enhanced proliferation and Runx2-associated osteogenic activity after AB treatment [13,14]. Purified polysaccharide fractions such as ABPB-4 also exhibit *in vitro* osteogenic activity, and fructooligosaccharide or polysaccharide components from AB have been shown to stimulate bone formation in experimental osteoporosis models [15,16]. In parallel, AB regulates the resorptive arm of remodeling, with prior studies demonstrating suppression of osteoclastogenesis and bone resorption through inhibition of RANKL signaling, as well as osteoprotective effects *in vivo* through regulation of the RANKL/RANK/OPG axis. A recent meta-analysis in osteoporotic rat models further reinforced its overall osteoprotective profile [17,18]. Together, these studies position AB as a bioactive factor with both anabolic and anti-resorptive potential, which is precisely the type of dual-function cue needed for functional bone regeneration.

Despite these advances, the two fields have largely progressed in parallel. Most studies of AB have focused on two-dimensional osteoblast or stromal-cell assays, phytochemical characterization, or systemic administration in animal models [19,20]. By contrast, most bone organoid studies have emphasized scaffold design, lineage induction, and three-dimensional assembly, without fully exploiting the regulatory potential of traditional osteoactive compounds [21,22]. As a result, it remains unclear whether AB can do more than improve isolated cellular osteogenesis. Specifically, it is unknown whether this herb can enhance organoid-level maturation, strengthen extracellular matrix mineralization within a three-dimensional bone-like construct, and ultimately translate into more effective repair of critical bone defects *in vivo* [23,24]. The central question of this study was whether AB could serve as an organoid-compatible osteoregenerative cue that enhances bone organoid maturation and improves defect repair *in vivo*.

In this study, we developed a translational strategy that integrates AB into a BMSC-derived bone organoid system for bone defect repair. We first evaluated the osteogenic effects of AB *in vitro* and then incorporated AB into bone organoid culture to generate AB-bone organoids. Their regenerative efficacy was subsequently tested in a bone defect model and the clinical relevance of AB was further explored in a retrospective fracture-healing cohort. Together, this work demonstrates that AB enhances bone organoid maturation and supports the preclinical potential of AB-based organoid engineering for bone defect repair.

## 2 Methods

### 2.1 Study overview

This study was designed to determine whether AB could function as an organoid-compatible osteoregenerative cue for bone defect repair. The workflow comprised four sequential components: dose screening of AB in bone marrow-derived mesenchymal stromal cells (BMSCs), generation and characterization of AB-bone organoids, evaluation of regenerative efficacy in a critical-sized calvarial defect model, and an exploratory retrospective clinical analysis of AB exposure in tibial fracture healing. All *in vitro* and *in vivo* experiments were performed independently at least three times unless otherwise specified. The study was conducted in accordance with institutional guidelines and approved by the Ethics Committee of Shanghai Sixth People's Hospital (DWLL2025-0263).

### 2.2 BMSC culture and AB treatment

Rat BMSCs were isolated from the femurs and tibias of 4–6-week-old male Sprague–Dawley rats as previously described and cultured in low-glucose Dulbecco's modified Eagle's medium supplemented with 10% fetal bovine serum (Gibco, Thermo Fisher Scientific, USA) and 1% penicillin–streptomycin (HyClone, USA) at 37°C in a humidified 5% CO_2_ incubator. Non-adherent cells were removed after 24 h, and the medium was changed every 2–3 days thereafter. Cells at passages 3–5 were used for all experiments.

AB was obtained from Beijing Medicinal Herbs Co., Ltd. (Beijing, China; batch number: 20230115). The raw botanical material was authenticated by macroscopic and microscopic examination according to the Pharmacopoeia of the People's Republic of China (2020 edition), with voucher specimens (No. AB-2023-BJ) deposited at the Herbarium of the Institute of Chinese Materia Medica, China Academy of Chinese Medical Sciences. Dried AB roots (500 g) were decocted twice with distilled water (1:10, w/v) at 100°C for 1 h each. The combined extracts were filtered, concentrated under reduced pressure, lyophilized, and stored at −20°C. The extraction yield was 18.5% (w/w). Before use, the extract was reconstituted in sterile PBS and filtered through a 0.22-μm membrane. For HPLC fingerprinting, the extract was analyzed on a C18 column using a gradient of acetonitrile and 0.1% phosphoric acid in water at 1.0 mL/min, with UV detection at 210 nm, and the total run time was 60 min. For osteogenic induction, BMSCs were cultured in osteogenic medium containing dexamethasone, β-glycerophosphate, and ascorbic acid.

### 2.3 Dose screening of AB *in vitro*

To define the most effective osteogenic concentration of AB, BMSCs were exposed to 0, 25, 50, 100, 200, and 400 μg/mL AB. Cell compatibility and proliferative activity were evaluated by live/dead staining and the Cell Counting Kit-8 (CCK-8, Dojindo, Japan) assay. Early osteogenic differentiation was assessed by alkaline phosphatase (ALP) staining (Sigma-Aldrich, USA), and matrix mineralization was assessed by Alizarin Red S (ARS) staining (Sigma-Aldrich, USA) after osteogenic induction. Total RNA was isolated for quantitative real-time PCR analysis of *Col1a1*, *Runx2*, *Opn*, and *Ocn*. On the basis of cell viability, osteogenic activity, and mineralization, 200 μg/mL was selected as the working concentration for subsequent organoid experiments.

### 2.4 Generation of bone organoids and AB-bone organoids

Bone organoids were generated by three-dimensional self-assembly of BMSCs. Briefly, single-cell suspensions of passage 3–5 BMSCs were seeded into ultra-low-attachment 24-well plates (1 × 10^6 cells per well) and cultured for 3 days to allow spontaneous formation of compact spheroids. The spheroids were then embedded in growth factor-reduced Matrigel (Corning, USA) in 96-well plates and cultured in osteogenic differentiation medium containing 10 mM β-glycerophosphate, 50 μg/mL ascorbic acid, and 100 nM dexamethasone for 28 days. To generate AB-bone organoids, AB (200 μg/mL) was added to the osteogenic medium throughout the maturation period, with medium refreshed every 2–3 days. Untreated organoids cultured under the same osteogenic conditions served as controls. Live/dead staining (Calcein-AM/PI), morphological assessment, ALP staining, western blotting, and osteogenic gene expression analyses were performed on day 7 after Matrigel embedding. Alizarin Red S staining and COL-I immunostaining were performed on day 21, unless otherwise indicated.

### 2.5 Histologic, molecular, and functional analyses

Viable and dead cells within organoids were visualized by live/dead staining using Calcein-AM (2 μM, Dojindo, Japan) and Propidium Iodide (PI, 4 μM, Sigma-Aldrich, USA). Bright-field imaging was used to document gross morphology, and organoid size was quantified by image analysis. Osteogenic maturation was evaluated by ALP staining, ARS staining, and COL-I staining. For immunofluorescence, organoids were fixed, permeabilized, and incubated with antibodies against Runx2; filamentous actin was visualized with phalloidin and nuclei with DAPI. To compare osteogenic performance across culture formats, monolayer BMSCs, untreated bone organoids, and AB-bone organoids were analyzed in parallel. Cell viability was measured by CCK-8 assay, protein expression of COL-I, Runx2, and OPN (Cell Signaling Technology, USA) was assessed by western blotting, and expression of Col1a1, Runx2, Opn, and Ocn was quantified by real-time PCR.

For western blotting, total protein was extracted in RIPA buffer, separated by SDS–PAGE, transferred to polyvinylidene fluoride membranes, and detected by enhanced chemiluminescence. For gene-expression analysis, total RNA was reverse-transcribed and relative transcript abundance was calculated using the 2^^−ΔΔCt^ method. The primer sequences used in the study are listed in Table S1.

### 2.6 ERK/MAPK pathway inhibition assay in BMSCs

BMSCs were assigned to four groups: control, AB, PD98059, and AB + PD98059. AB was applied at 200 μg/mL. PD98059 (Sigma-Aldrich, St. Louis, MO, USA), a selective ERK/MAPK pathway inhibitor, was used at 20 μM, with 1 h pretreatment before AB administration in the combination group. Vehicle concentrations were kept identical among groups. For ERK/MAPK activation analysis, protein lysates were collected 30 min after treatment for western blotting of phosphorylated and total ERK1/2 (Cell Signaling Technology, USA). For osteogenic evaluation, cells were cultured under osteogenic induction with the indicated treatments. ALP staining was performed on day 7 and Alizarin Red S staining on day 21. Stained images were quantified using ImageJ under identical acquisition and analysis parameters.

### 2.7 Critical-sized calvarial defect model

Adult male Sprague–Dawley rats aged 8 weeks and weighing 250–300 g were used for the calvarial defect experiment. Animals were anesthetized with intraperitoneal pentobarbital sodium (40 mg/kg), and a critical-sized calvarial defect model was established under sterile conditions. After surgical exposure of the calvarium, a standardized full-thickness 5-mm defect was created under saline irrigation while preserving the underlying dura. Animals were randomly assigned to one of three groups (n = 5 per group): defect only, bone organoids, or AB-bone organoids. The corresponding constructs were implanted directly into the defect bed, and the overlying soft tissues were closed in layers. Postoperative care was provided according to institutional protocols. Animals were euthanized at the predefined endpoint for radiographic and histologic evaluation.

### 2.8 Micro-CT, histology, and immunohistochemistry

At 12 weeks after implantation, calvarial specimens were harvested and analyzed by micro-CT. Three-dimensional reconstructions were generated to assess the extent of defect bridging and mineralized tissue formation. Quantitative analysis within the defect region included bone volume fraction (BV/TV), bone mineral density (BMD), trabecular thickness (Tb.Th), trabecular number (Tb.N), and trabecular separation (Tb.Sp). After micro-CT scanning, specimens were fixed, decalcified, paraffin-embedded, and sectioned. Hematoxylin and eosin (H&E) staining and Masson's trichrome staining were performed to evaluate tissue architecture, new bone formation, and collagen deposition. Immunohistochemical staining for COL-I, OPN, and OCN was used to assess bone matrix deposition within the regenerative area. Histologic and immunohistochemical analyses were performed using standardized imaging parameters and quantified by blinded assessors.

### 2.9 Retrospective clinical cohort

The clinical cohort was included as an exploratory analysis to evaluate whether the experimentally observed osteoregenerative effects of AB translate to human fracture healing. This retrospective study was conducted at Taizhou Hospital of Traditional Chinese Medicine (Jiangsu, China) in accordance with the Declaration of Helsinki and Good Clinical Practice, with approval from the institutional ethics board (HJSW-25071605). Consecutive patients with tibial fractures who underwent surgical treatment between January 2022 and December 2024 were screened. The objective was to examine whether postoperative AB administration was associated with improved fracture-healing outcomes as compared with standard postoperative management. Because only anonymized retrospective clinical data were analyzed, the requirement for additional written informed consent was waived by the institutional ethics board.

Patients were eligible if they were 18 to 70 years of age, had a radiographically confirmed tibial fracture treated with operative fixation, had complete baseline and follow-up data, and had been followed for at least 12 months. Patients were excluded if they had a pathological fracture, previous fracture or surgery involving the same tibia, active infection, severe systemic disease likely to affect fracture repair, concurrent use of medications known to substantially alter bone metabolism, incomplete imaging records, or loss to follow-up. Of 126 screened patients, 40 met the eligibility criteria and were included in the final analysis, with 20 in the AB group and 20 in the control group.

Exposure status was determined from documented postoperative management. Patients in the AB group received oral AB beginning 1 month after surgery for 6 weeks at 25 g twice daily. Patients in the control group received standard postoperative care without AB. Because of the retrospective design, treatment allocation was based on actual clinical practice rather than random assignment. Apart from AB exposure, all patients received routine perioperative management, including wound care, rehabilitation guidance, and serial radiographic follow-up.

Baseline variables extracted from the electronic medical record included age, sex, body-mass index, smoking status, diabetes, fracture side, operative duration, intraoperative blood loss, hospital stay, and baseline pain and lower-extremity function. Standard anteroposterior and lateral radiographs obtained during follow-up were reviewed at 1, 3, 6, and 12 months after treatment initiation. The primary outcome was radiographic healing assessed with the modified Radiographic Union Scale for Tibial fractures (mRUST); healing time was also recorded as an imaging-based indicator of recovery. Secondary outcomes were pain assessed by the visual analogue scale (VAS) and function assessed by the Lower Extremity Functional Scale (LEFS) at 6 and 12 months postoperatively. Radiographs were independently evaluated by orthopaedic assessors who were unaware of treatment exposure, and disagreements were resolved by consensus.

### 2.10 Statistical analysis

Continuous variables are presented as mean ± standard deviation, and categorical variables as counts and percentages. For *in vitro* and animal experiments, comparisons between two groups were performed with the unpaired Student's t-test, and comparisons among multiple groups were performed with one-way analysis of variance followed by post hoc multiple-comparison testing. For the clinical cohort, between-group differences in continuous variables were assessed with the independent-samples *t*-test, and categorical variables were compared with the chi-square test or Fisher's exact test, as appropriate. Longitudinal radiographic outcomes were analyzed with repeated-measures analysis. All tests were two-sided, and a P value of less than 0.05 was considered to indicate statistical significance.

## 3 Results

### 3.1 AB concentration screening and its osteogenic effects on BMSCs

The AB preparation was authenticated according to the Pharmacopoeia of the People's Republic of China (2020 edition) and characterized by a multi-peak HPLC fingerprint, establishing its botanical identity and batch traceability (Fig. S1). Following this characterization, BMSCs were exposed to graded concentrations of AB (0–400 μg/mL) to define the most effective osteogenic dose *in vitro*. Live/dead staining demonstrated good cytocompatibility across the low- and intermediate-dose groups, whereas the highest dose showed a relative reduction in viable cell density (Fig. 1A). Consistent with this pattern, CCK-8 analysis revealed a dose-dependent increase in cell viability and proliferative activity from 0 to 200 μg/mL, with 200 μg/mL representing the most effective concentration. At 400 μg/mL, the proliferative benefit was attenuated, indicating that the osteopromotive window of AB was concentration dependent rather than linearly cumulative (Fig. 1D).

**Fig. 1:**
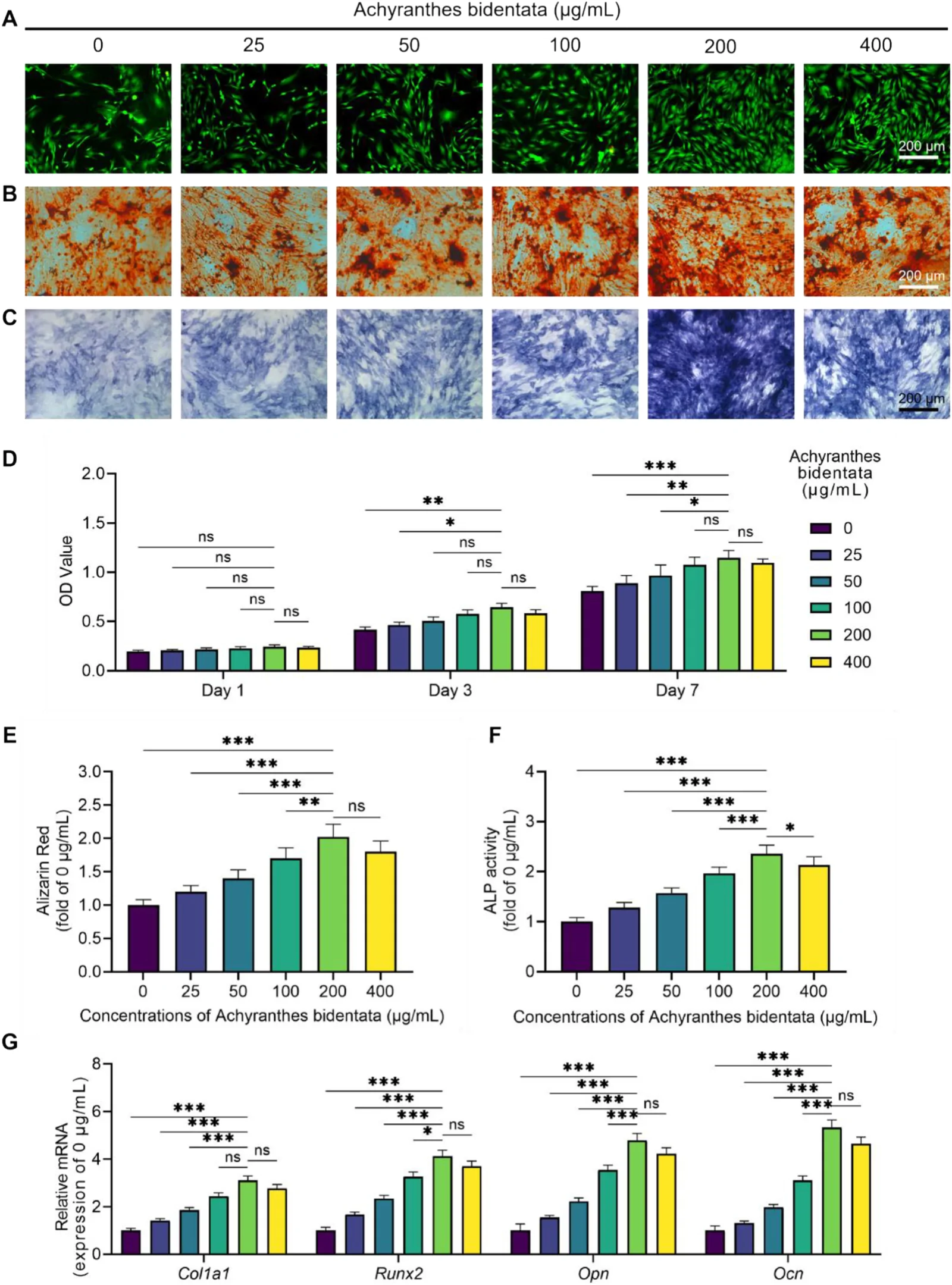
*Achyranthes bidentata* (AB) promotes proliferation and osteogenic differentiation. (A–C) Live/dead staining (day 7), alkaline phosphatase (ALP) staining (day 7), and Alizarin Red S (ARS) staining (day 21) of BMSCs treated with AB (0–400 μg/mL). (D–F) Quantification of CCK-8 (D), ARS (E), and ALP (F) showing dose-dependent enhancement with peak activity at 200 μg/mL (G) qPCR of osteogenic genes (*Col1a1*, *Runx2*, *Opn*, and *Ocn*) at day 7. Data are mean ± SD; n = 5; ns = no significance, *P < 0.05, **P < 0.01, ***P < 0.001.

Functional staining further supported the anabolic effects of AB. Both ALP and ARS staining intensified progressively with AB treatment and reached maximal levels at 200 μg/mL, indicating enhanced early osteogenic differentiation and matrix mineralization (Fig. 1B, C, 1E, 1F). At the transcriptional level, qPCR showed that AB markedly upregulated the osteogenic program, as reflected by increased expression of *Col1a1*, *Runx2*, *Opn*, and *Ocn* at day 7. Together, these findings identify 200 μg/mL as the selected working concentration within the tested range in monolayer BMSCs, providing the basis for its subsequent use in the proof-of-concept organoid experiments (Fig. 1G).

### 3.2 ERK/MAPK signaling partially contributes to the pro-osteogenic effect of AB in BMSCs

To investigate the involvement of ERK/MAPK signaling in AB-induced osteogenesis, BMSCs were treated with AB in the presence or absence of the ERK/MAPK inhibitor PD98059. AB markedly enhanced ALP activity and mineralized matrix formation, whereas PD98059 attenuated both effects, as shown by ALP staining, Alizarin Red S staining, and corresponding quantitative analyses (Fig. 2A). Western blotting further showed that AB increased ERK1/2 phosphorylation without markedly affecting total ERK1/2 expression, while PD98059 partially suppressed AB-induced ERK1/2 activation, as confirmed by quantification of the p-ERK1/2/ERK1/2 ratio (Fig. 2B). These results indicate that ERK/MAPK signaling partially contributes to the pro-osteogenic effect of AB, as demonstrated by the inhibitory effect of PD98059 (Fig. 2C).

**Fig. 2:**
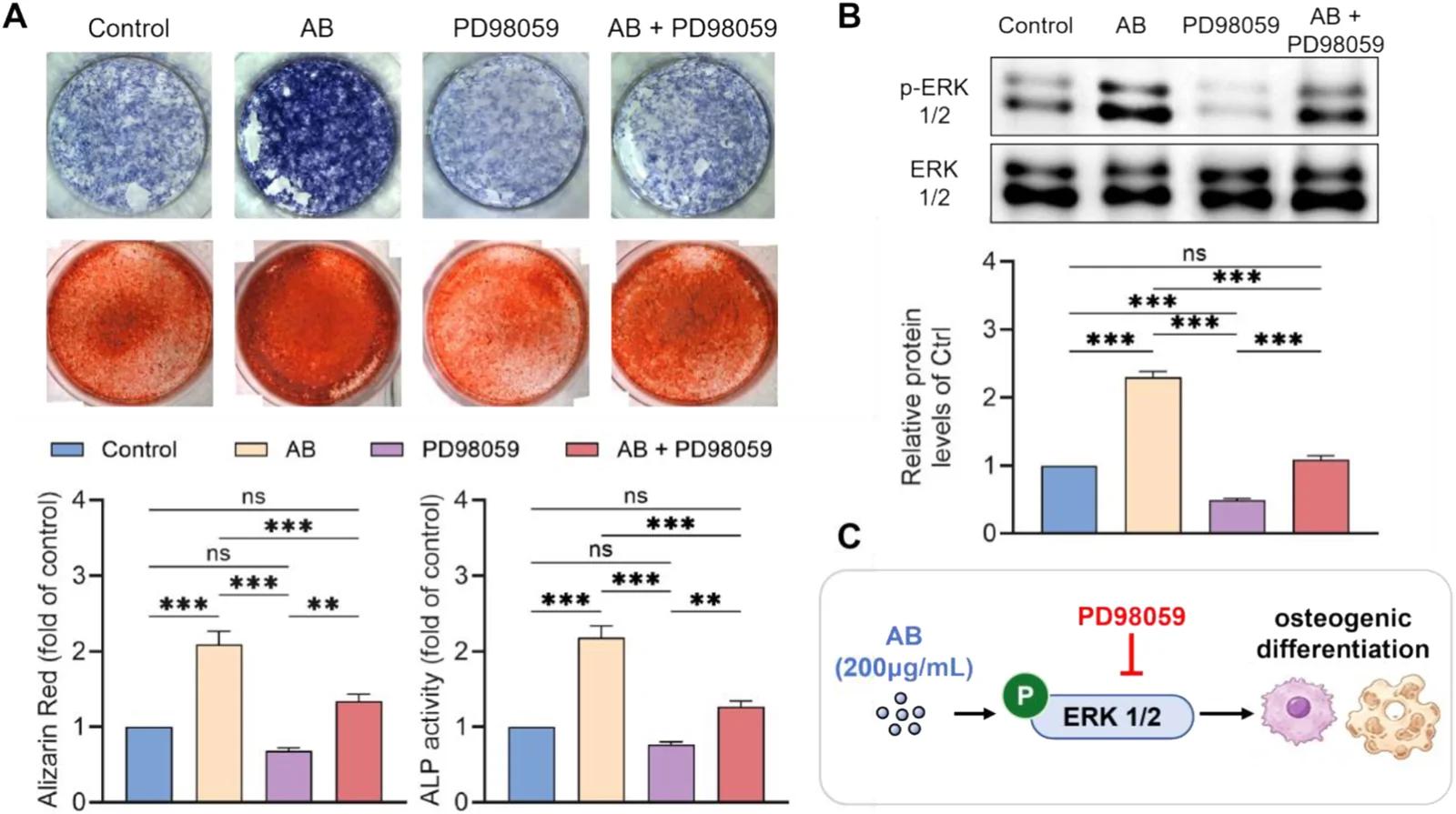
ERK/MAPK signaling partially contributes to the pro-osteogenic effect of AB in BMSCs. (A) Representative ALP staining (day 7) and ARS staining (day 21) with quantitative analysis in BMSCs treated with control, AB (200 μg/mL), PD98059 (20 μM), or AB + PD98059. (B) Western blot analysis of phosphorylated and total ERK1/2 with quantification of the p-ERK1/2/ERK1/2 ratio. (C) Schematic illustration of the potential contribution of ERK/MAPK signaling to AB-enhanced osteogenic differentiation in BMSCs. Data are presented as mean ± SD; ns, not significant; **P < 0.01, ***P < 0.001.

### 3.3 AB accelerates bone organoid maturation

We next investigated whether AB could promote the maturation of BMSC-derived bone organoids. As shown in Fig. 3A, BMSC spheroids were generated and embedded in Matrigel, then cultured under osteogenic conditions in the presence or absence of 200 μg/mL AB. Live/dead staining (Calcein-AM/PI) revealed that both groups remained viable, but AB-treated organoids displayed a more widespread Calcein-AM-positive signal and reduced PI incorporation, suggesting improved viability distribution (Fig. 3B). Because large spheroid-based constructs may be vulnerable to diffusion limitation, organoid morphology and viability were evaluated before downstream functional analyses. While gross morphological observations revealed no discernible differences between the two groups (Fig. S2A), rigorous quantitative analysis (Fig. S2B) demonstrated that AB-treated organoids exhibited a larger cross-sectional area, suggesting that AB may facilitate accelerated tissue assembly and volumetric growth. Histological and matrix analyses confirmed these differences. Compared with untreated organoids, AB-bone organoids exhibited stronger ARS, ALP, and COL-I staining, indicating enhanced calcium deposition, osteogenic enzyme activity, and extracellular matrix production (Fig. 3C). Quantitative analysis further showed significantly increased ARS, ALP, and COL-I signals in the AB-treated group (Fig. 3D). Immunofluorescence staining demonstrated more organized F-actin architecture and stronger Runx2 expression in AB-bone organoids (Fig. 3E), consistent with heightened osteogenic lineage activation. Collectively, these findings indicate that AB not only supports organoid survival, but also accelerates the maturation of bone organoids toward a more mineralized and osteogenic phenotype.

**Fig. 3:**
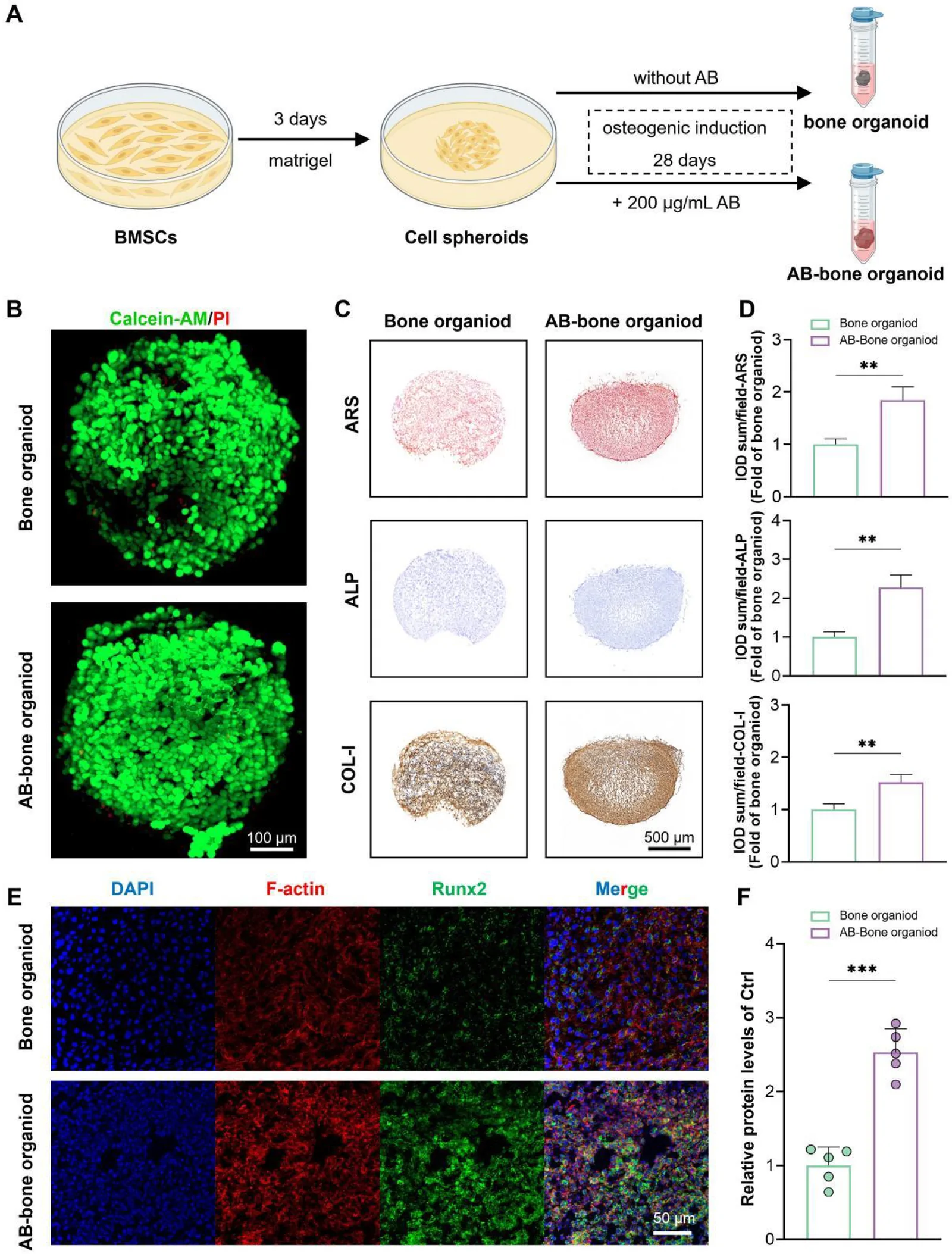
*Achyranthes bidentata* (AB) accelerates bone organoid maturation. (A) Schematic of bone organoid generation with or without AB treatment (200 μg/mL). BMSCs were self-aggregated for 3 days, embedded in Matrigel, and cultured in osteogenic medium for up to 28 days. (B, C) Live/dead staining (Calcein-AM/PI, day 7) and histology (ARS, ALP, COL-I) of control and AB-bone organoids. (D) Quantification of ARS (day 21), ALP (day 7), and COL-I (day 21) revealed enhanced mineralization and matrix deposition in AB-bone organoids. (E) Representative immunofluorescence images of F-actin (red) and Runx2 (green) in organoids on day 21, with quantitative analysis of Runx2 fluorescence intensity. Data are mean ± SD; **P < 0.01, ***P < 0.001.

### 3.4 AB-bone organoids exhibit superior osteogenic output *in vitro*

To determine whether AB-bone organoids provided a functional advantage beyond standard monolayer culture and untreated bone organoids, we compared three conditions: monolayer BMSCs, bone organoids, and AB-bone organoids. Both ALP and ARS staining revealed a stepwise enhancement in osteogenic output across the three groups, with the weakest staining observed in monolayer cultures, intermediate staining in bone organoids, and the strongest staining in AB-bone organoids (Fig. 4A). Quantitative analyses confirmed this hierarchy, demonstrating that AB-bone organoids achieved the highest levels of enzymatic osteogenic activity and matrix mineralization (Fig. 4C). This functional superiority was accompanied by improved cellular performance. At day 7, CCK-8 assay showed higher viability in AB-bone organoids than in either monolayer cultures or untreated organoids (Fig. 4D). Western blot analysis and quantification further demonstrated increased expression of COL-I, Runx2, and OPN in the AB-bone organoid group, indicating more advanced osteoblastic maturation (Fig. 4B). Consistent with these protein-level findings, qPCR confirmed significant upregulation of *Col1a1*, *Runx2*, *Opn*, and *Ocn* in AB-bone organoids (Fig. 4E). Together, these results indicate that AB integration establishes a more potent osteogenic niche, enabling bone organoids to outperform both conventional 2D cultures and untreated 3D constructs.

**Fig. 4:**
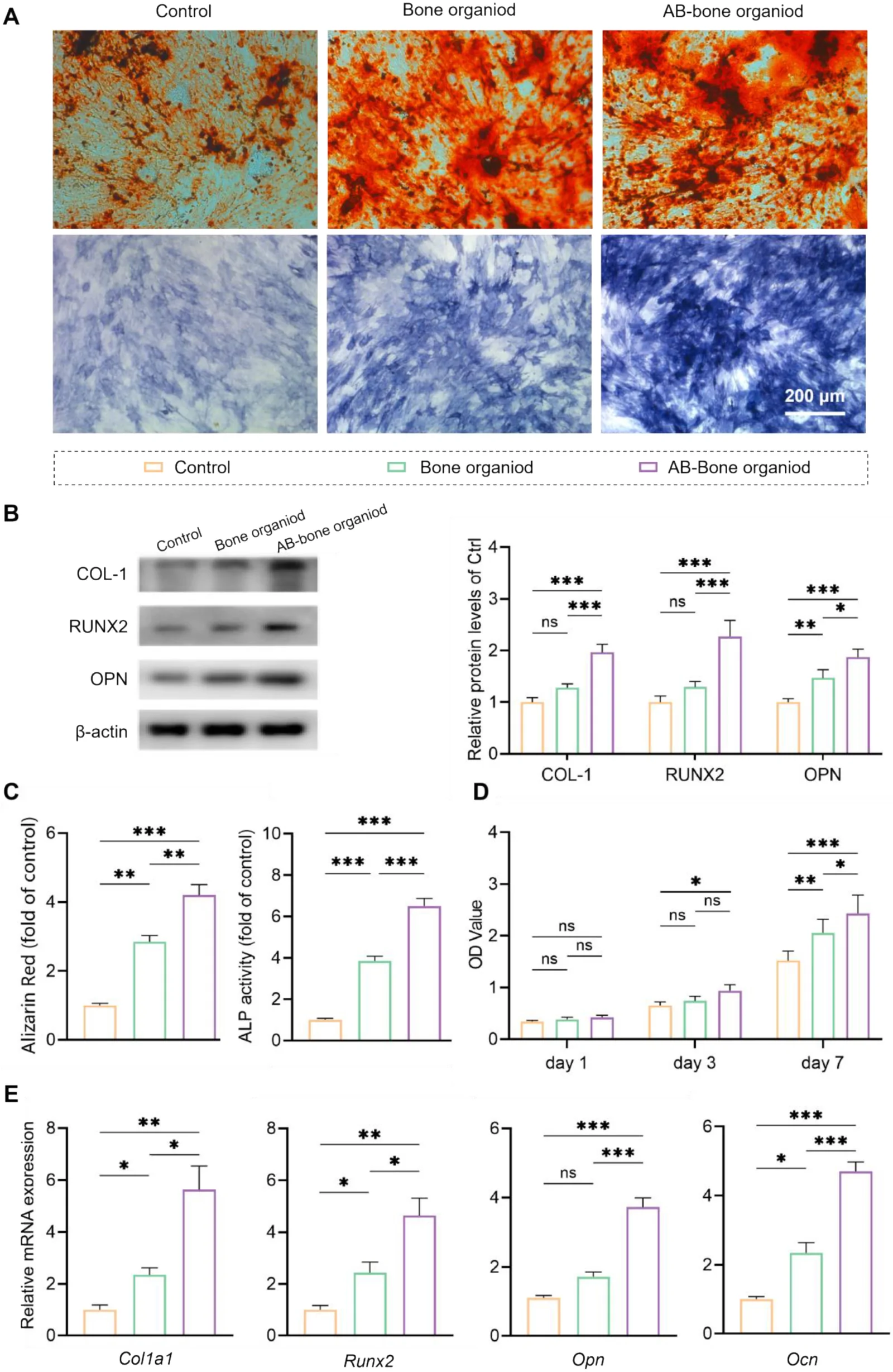
*Achyranthes bidentata* (AB)-bone organoids exhibit superior osteogenic output. (A) ARS and ALP staining of monolayer, bone organoid, and AB-bone organoid cultures. (B) Western blot of COL-I, Runx2, and OPN, along with quantitative analysis of relative protein levels. (C) Quantitative analysis of ARS (day 21) and ALP staining (day 7) intensity. (D) CCK-8 proliferation assay showing enhanced cell viability in AB-bone organoids at day 7. (E) Quantitative analysis of osteogenic marker gene expression (*Col1a1*, *Runx2*, *Opn*, and *Ocn*). Data are mean ± SD; ns = no significance, *P < 0.05, **P < 0.01, ***P < 0.001.

### 3.5 AB-bone organoids enhance calvarial defect repair *in vivo*

We next assessed whether the *in vitro* advantages of AB-bone organoids translated into improved bone regeneration *in vivo* (Fig. 5A). In a critical-sized calvarial defect model, animals treated with AB-bone organoids showed more substantial new bone formation than those receiving untreated bone organoids or defect-only controls. Representative micro-CT reconstructions and cross-sectional images at 12 weeks revealed more extensive defect bridging and denser mineralized tissue in the AB-bone organoid group (Fig. 5B). Quantitative analysis confirmed these structural improvements. The AB-bone organoid group showed the most favorable bone microarchitecture, with increased BV/TV, BMD, Tb.Th, and Tb.N, together with reduced Tb.Sp, relative to the other groups. Untreated bone organoids also improved repair compared with empty defects, but their effect was consistently less pronounced than that of AB-bone organoids (Fig. 5C). Histological analyses further corroborated these radiographic findings. H&E and Masson's trichrome staining demonstrated significantly greater bone fill and more organized collagen matrix deposition within the defects treated with AB-bone organoids, compared to the sparse connective tissue observed in the control groups (Fig. 5D–S3). To further interrogate osteogenic activation at the molecular level, we performed immunohistochemical staining for COL-I, OPN, and OCN. The AB-bone organoid group showed more abundant COL-I-positive extracellular matrix deposition within the defect area, suggesting a more mature and active osteogenic reparative niche (Fig. 5E–S4). A similar pattern was observed for OPN and OCN (Fig. S4). Collectively, these data demonstrate that AB functionalization enhances the regenerative efficacy of bone organoids and promotes superior structural restoration of calvarial defects.

**Fig. 5:**
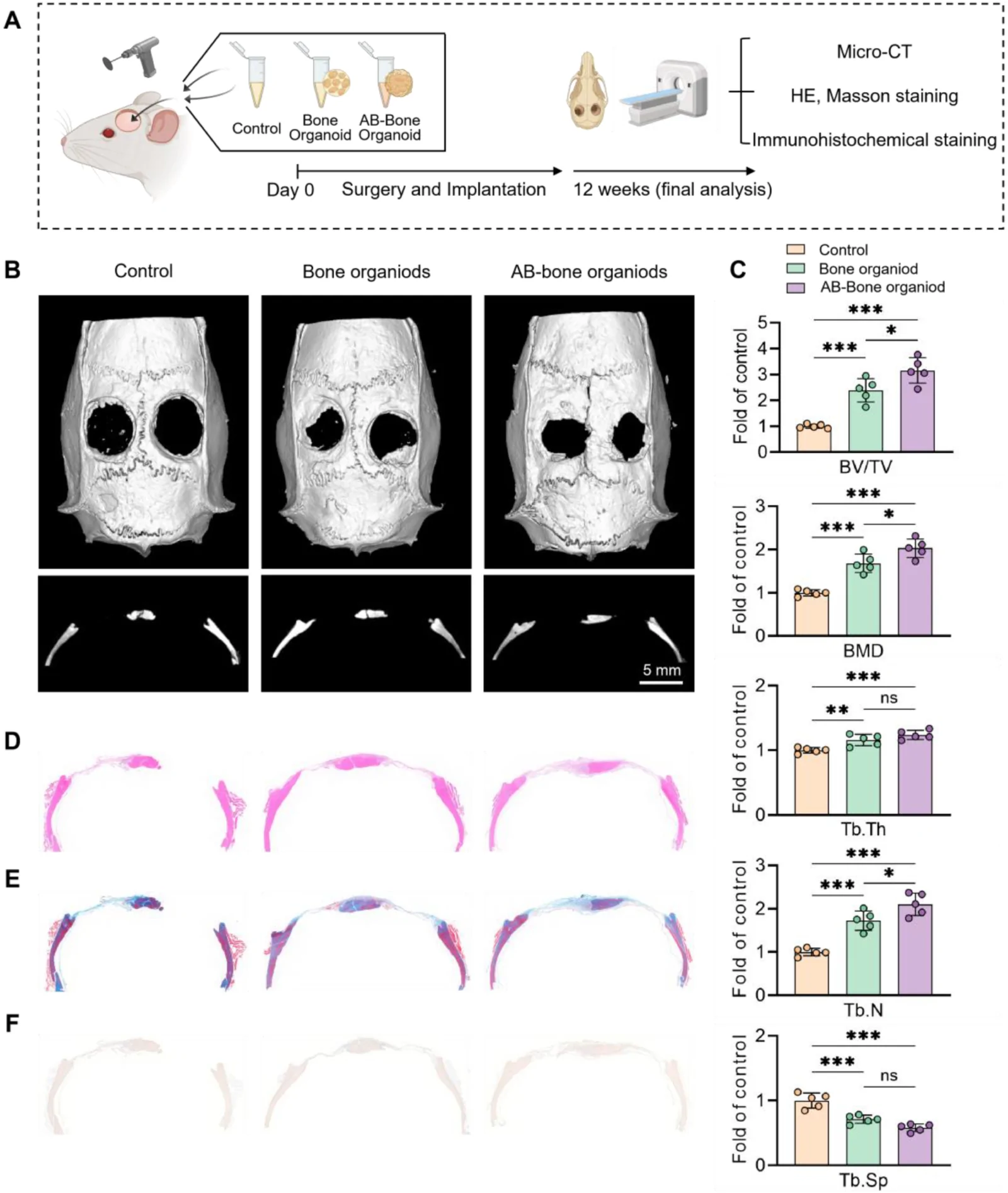
*Achyranthes bidentata* (AB)-bone organoids enhance calvarial defect repair. (A) Schematic of critical-sized calvarial defect surgery, organoid implantation and subsequent evaluation. (B) Representative micro-CT reconstructions and cross-sections at 12 weeks. (C) Quantitative analysis of bone microarchitecture parameters (BV/TV, BMD, Tb.Th, Tb.N, Tb.Sp). (D) Representative histological images of the defect site stained with Hematoxylin and Eosin (H&E) and Masson's Trichrome at 12 weeks. (E) Immunohistochemical staining of COL-I within the regenerative area. The AB-bone organoid group demonstrates a significantly higher density of COL-I-positive extracellular matrix deposition. Data are mean ± SD; n = 5; ns = no significance, *P < 0.05, **P < 0.01, ***P < 0.001.

### 3.6 Clinical cohort enrollment and baseline characteristics

The clinical study flow is summarized in Fig. 6. Consecutive patients with tibial fractures treated between January 2022 and December 2024 were screened according to the predefined eligibility criteria. A total of 40 eligible patients were included in the final analysis, with 20 assigned to the AB group and 20 to the control group. The baseline demographic and clinical characteristics were well balanced between the two study cohorts (Table 1). The mean age, sex distribution, and body-mass index were comparable in the two groups. There were no significant between-group differences in medical comorbidities (smoking and diabetes) or injury characteristics (fracture side). Similarly, perioperative parameters, including operative time, blood loss, and duration of hospital stay, were similar across both groups. At enrollment, baseline symptom intensity and functional status, as measured by VAS and LEFS scores, were also well matched. Overall, these data indicate that the two groups were highly comparable at baseline, providing a rigorous foundation for evaluating the efficacy of postoperative AB administration.

**Fig. 6:**
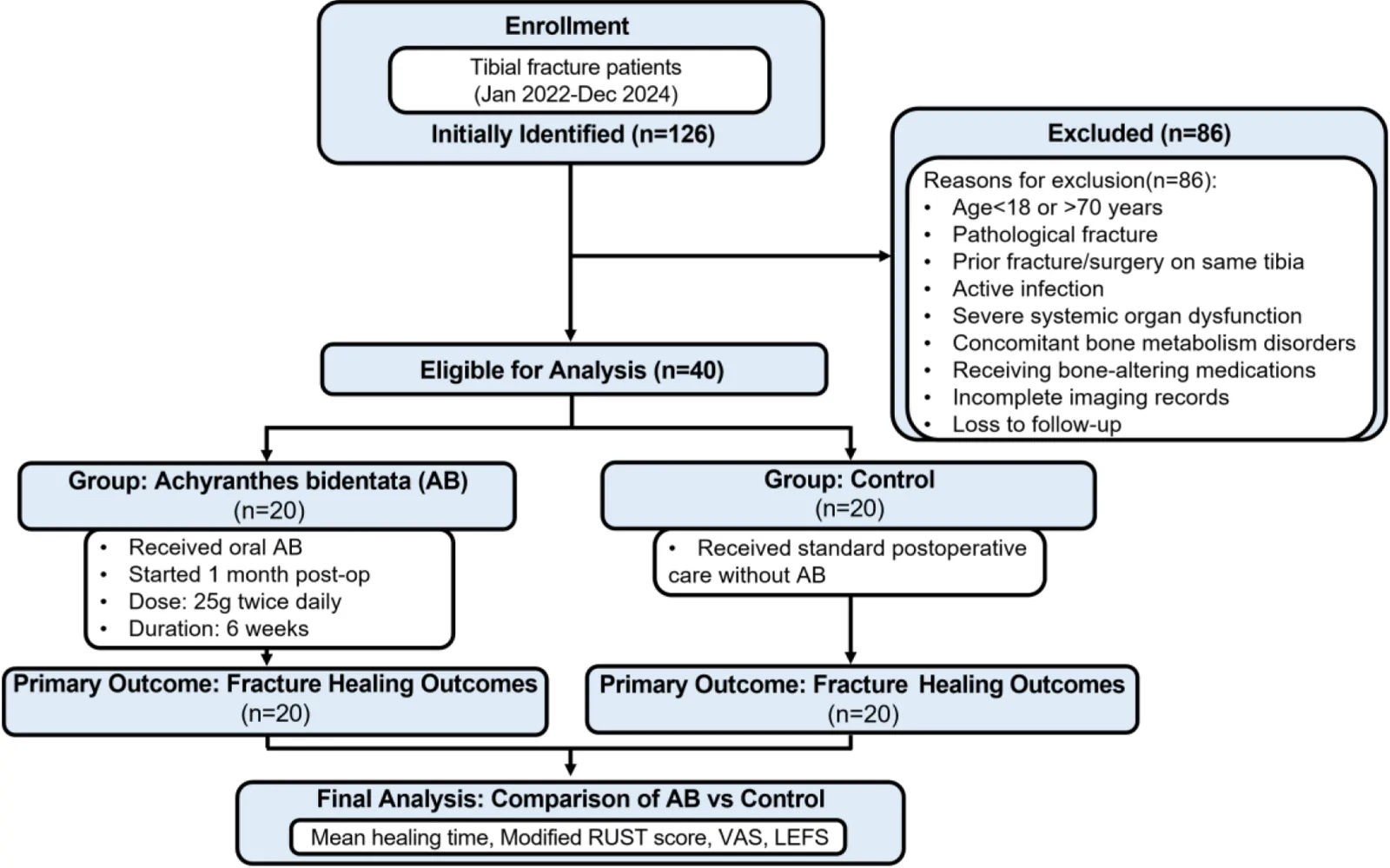
Clinical study flow diagram.

**Table 1:** Participant demographics and clinical characteristics. AB, *Achyranthes bidentata*; BMI, body mass index; VAS, visual analogue scale; and LEFS, lower extremity functional scale.

Characteristic	AB group (n = 20)	Control group (n = 20)	P value
Age (years)	44.3 ± 12.6 (21–68)	45.7 ± 11.9 (23–70)	0.720
Sex (Male/Female)	13/7 (65%/35%)	14/6 (70%/30%)	1.000
BMI (kg/m^2^)	24.6 ± 3.1 (19.8–30.2)	24.2 ± 3.4 (18.9–29.7)	0.700
Smoking, n (%)	6 (30%)	7 (35%)	1.000
Diabetes, n (%)	2 (10%)	3 (15%)	1.000
Side (Left/Right)	11/9 (55%/45%)	10/10 (50%/50%)	1.000
Operative time (min)	86.5 ± 18.2 (60–125)	89.3 ± 20.6 (65–130)	0.651
Blood loss (ml)	142 ± 55 (60–260)	148 ± 63 (70–280)	0.750
Hospital stay (days)	8.6 ± 2.7 (5–14)	8.7 ± 3.0 (5–15)	0.912
VAS before surgery	7.3 ± 1.1	7.5 ± 1.0	0.551
LEFS before surgery	18.6 ± 6.2	19.1 ± 5.8	0.794

### 3.7 Exploratory clinical association of postoperative AB administration with fracture-healing outcomes

Postoperative AB administration was associated with shorter radiographic healing time compared with standard postoperative management in this exploratory retrospective cohort (Table 2). The mean healing time was substantially shorter in the AB group (3.8 ± 0.7 vs. 4.9 ± 0.9 months; P < 0.001), indicating that postoperative AB administration was associated with shorter radiographic healing time. This radiographic advantage was further reflected by the modified RUST score. As shown in Fig. 7A, mRUST scores were higher in the AB group at both 6 and 12 months, indicating more rapid progression of cortical bridging and union. At the final follow-up, the AB administration group demonstrated a significantly higher mRUST score than the control group (14.1 ± 1.2 vs. 12.3 ± 1.5; P < 0.001). Although the proportion of patients achieving union by 6 months was numerically greater in the AB group, the difference did not reach statistical significance (P = 0.236), suggesting that AB's primary benefit lies in the speed and quality of the maturation process. Consistent with the radiographic findings, the AB group showed greater improvement in pain and lower-extremity function. As shown in Fig. 7B, at the final assessment, patients treated with AB reported significantly lower pain intensity (VAS, 1.3 ± 0.8 vs. 2.6 ± 1.1; P < 0.001), and superior lower-extremity function (LEFS, 72.5 ± 6.4 vs. 65.2 ± 7.3; P = 0.0018). Serial radiographs further confirmed earlier callus formation and advanced remodeling in AB-treated patients (Fig. 7C–S5, and S6). Collectively, postoperative AB administration was associated with higher mRUST scores, lower pain scores, and better functional outcomes after tibial fracture fixation.

**Table 2:** Comparison of radiographic and clinical outcomes between the *Achyranthes bidentata* (AB) group and the control group. mRUST, modified radiographic union scale for tibial fractures; VAS, visual analogue scale; and LEFS, lower extremity functional scale.

Variable	AB group (n = 20)	Control group (n = 20)	P value
Mean healing time (months)	3.8 ± 0.7	4.9 ± 0.9	< 0.001
mRUST score	14.1 ± 1.2	12.3 ± 1.5	< 0.001
VAS at last follow-up (12 months)	1.3 ± 0.8	2.6 ± 1.1	< 0.001
LEFS at last follow-up (12 months)	72.5 ± 6.4	65.2 ± 7.3	0.0018
Union at 6 months, n (%)	18 (90%)	14 (70%)	0.236

**Fig. 7:**
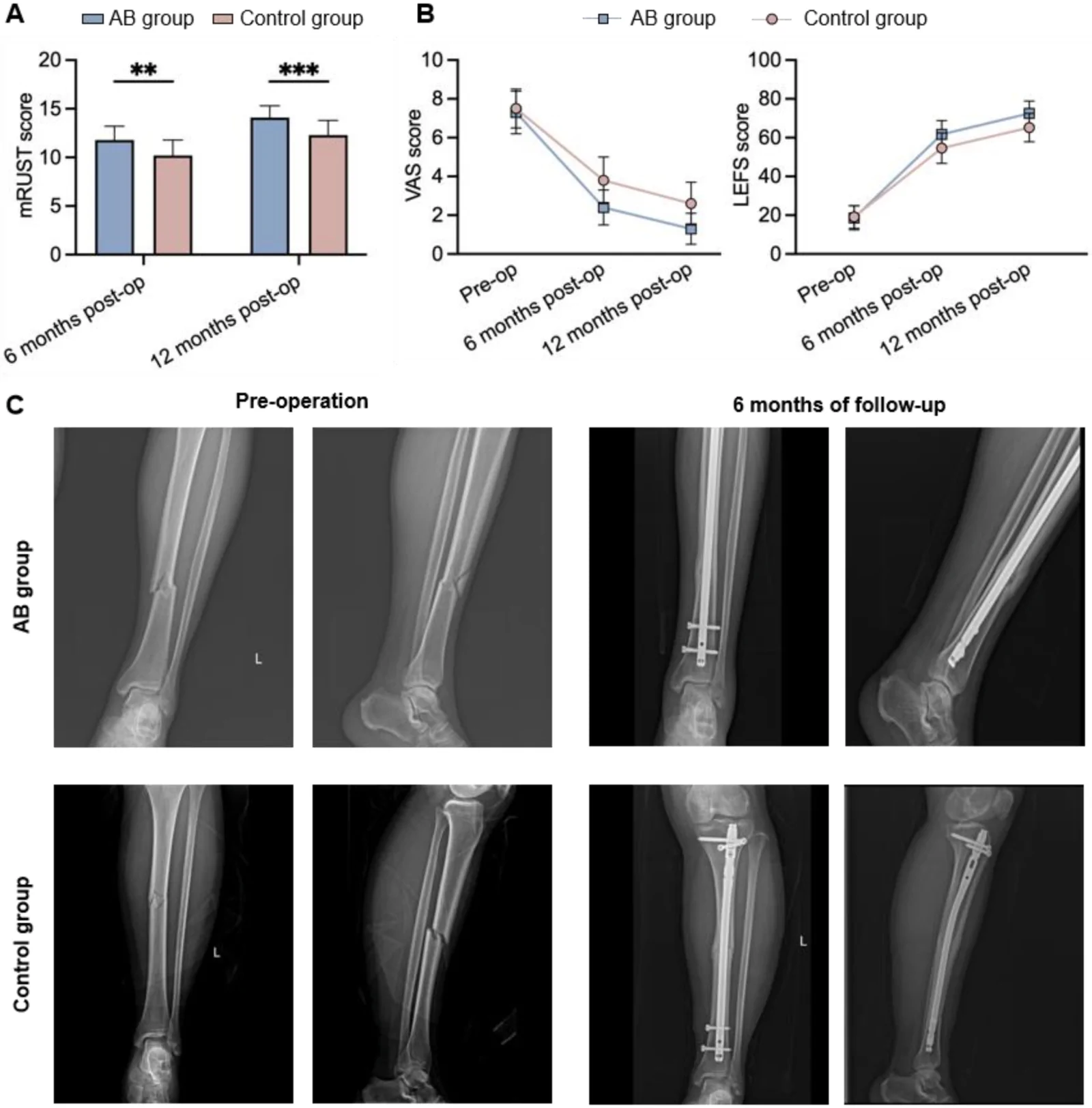
Exploratory association between postoperative *Achyranthes bidentata* (AB) administration and tibial fracture-healing outcomes. (A) Modified RUST scores at 6 and 12 months. AB group shows faster radiographic union. (B) VAS and LEFS scores show AB group exhibits superior pain relief and functional recovery. (C) Representative serial radiographs showing accelerated callus formation and remodeling in AB-treated patients. post-op, postoperative; Data are mean ± SD. **P < 0.01, ***P < 0.001.

## 4 Discussion

Repair of large bone defects remains limited by a persistent translational gap: many current approaches can stimulate osteogenesis, but few can simultaneously provide a living osteogenic unit, sustain three-dimensional matrix maturation, and remain compatible with clinically relevant deployment [25,26]. In this study, we address this gap by showing that AB can function not simply as a soluble osteogenic stimulant, but as an organoid-compatible bioactive cue that upgrades the regenerative state of bone organoids. This distinction is central. Previous studies have established the osteoprotective pharmacology of AB, whereas organoid-based strategies have emphasized the value of self-organized three-dimensional microtissues. Yet these two lines of research have largely advanced in parallel. By integrating AB into a BMSC-derived bone organoid platform and extending the work from *in vitro* screening to defect repair and clinical observation, our study connects phytopharmacology with regenerative organoid engineering and offers a new conceptual route for bone repair [27,28].

This work also moves beyond the conventional AB literature. Prior studies have mainly focused on phytochemical characterization, isolated fractions such as polysaccharides, saponins, and ketosteroids, or systemic administration in models of bone loss and osteonecrosis. These studies collectively support an osteoprotective role for AB, including promotion of osteoblastic activity, preservation of bone microarchitecture, and modulation of bone remodeling. However, AB has generally been treated as an extract acting on bone tissues from the outside. Here, by contrast, AB is incorporated into a living engineered construct, such that the therapeutic unit is no longer AB alone, nor cells alone, but an AB-functionalized bone organoid with pre-established osteogenic organization. This shift from pharmacologic supplementation to biologically instructed tissue engineering represents a substantive advance over both domestic and international studies. It should be noted that the 200 μg/mL concentration used in the organoid experiments was selected based on dose–response screening in monolayer BMSCs and should therefore be regarded as a working concentration rather than an optimized dose for the three-dimensional system. Future studies will require organoid-specific dose–response validation to account for matrix-associated diffusion and spatial exposure gradients.

A further important contribution is that our study extends AB biology from conventional two-dimensional assays to organoid-level maturation. Earlier work demonstrated that AB-related components enhance osteoblast proliferation, alkaline phosphatase activity, Runx2 expression, and matrix mineralization, but these readouts were largely confined to monolayer systems or simplified *in vitro* settings [29,30]. In the present study, these osteogenic effects were not merely reproduced but amplified within a three-dimensional self-organized context. AB-bone organoids exhibited greater ALP and ARS activity, stronger COL-I deposition, and higher Runx2 expression than untreated organoids, indicating that AB promotes not only cellular osteogenesis but also the transition from cell aggregate to a more mature bone-like microtissue. At the signaling level, our inhibitor-based experiments in monolayer BMSCs support a potential contribution of ERK/MAPK signaling to the pro-osteogenic activity of AB. AB increased ERK1/2 phosphorylation, whereas PD98059 partially inhibited ERK1/2 activation and attenuated AB-enhanced ALP activity and mineralized matrix formation. These findings suggest that ERK/MAPK signaling partially contributes to the pro-osteogenic effect of AB in BMSCs. Moreover, the incomplete inhibitory effect of PD98059 indicates that ERK/MAPK does not fully account for the biological activity of AB. Considering the multi-component nature of AB, other pathways, such as BMP/Smad, Wnt/β-catenin, RANKL/OPG, oxidative-stress regulation, and inflammatory modulation, may also participate in AB-mediated osteogenic maturation [31,32].

The *in vivo* findings further underscore the significance of this strategy. Compared with untreated organoids and empty defects, AB-bone organoids produced more extensive new bone formation, better defect bridging, more organized collagen deposition, and stronger osteogenic marker expression [33,34]. These data are consistent with prior reports showing that AB extracts improve bone formation and microarchitectural parameters *in vivo*, but they also go beyond them in an important respect: AB was not administered merely as a systemic or local extract, but embedded within a localized, living bone-like regenerative unit. This is particularly relevant for focal bone reconstruction, where successful repair depends not only on osteogenic stimulation, but also on matrix organization, tissue integration, and the establishment of a favorable reparative niche. Our findings therefore suggest that AB functionalization improves both the quantity and quality of newly formed bone.

Another strength of this study is its multi-level experimental design spanning *in vitro*, *in vivo*, and exploratory clinical investigations. Many regenerative studies end with *in vitro* staining or proof-of-concept histology in animals. Here, we structured the work across four levels: concentration screening, organoid maturation, calvarial defect repair, and a postoperative clinical cohort. The retrospective clinical cohort provides complementary evidence supporting the potential association between postoperative AB administration and fracture-healing outcomes, thereby providing clinical context for our preclinical findings. Although the clinical analysis evaluated postoperative AB administration rather than the organoid platform itself, the directionally consistent findings across cell culture, organoid maturation, animal regeneration, and human fracture healing strengthen the overall translational relevance of this study [35].

It is essential to distinguish between the animal and clinical components of this study. The animal experiment evaluated localized implantation of AB-preconditioned bone organoids into a critical-sized calvarial defect, representing a cell-therapy approach with localized AB exposure. In contrast, the clinical cohort evaluated systemic oral administration of AB decoction in patients with tibial fractures, representing a pharmacological intervention with systemic exposure, first-pass metabolism, and entirely different pharmacokinetics. These are fundamentally different interventions with different mechanisms, dosing regimens, and tissue exposures. The clinical findings therefore provide only indirect, associative support for the osteoregenerative relevance of AB as a botanical factor; they do not constitute evidence for the clinical feasibility of AB-bone organoid implantation, which would require dedicated clinical trials evaluating the organoid construct itself.

Several limitations should be acknowledged. First, the calvarial defect model is non-load-bearing and therefore does not fully reproduce the biomechanical environment of long-bone or segmental defects [36]. Second, although osteoangiogenic coupling is increasingly recognized as essential for functional bone regeneration, vascularization was not directly quantified in the present study [37]. The present constructs represent BMSC-derived osteogenic bone organoids rather than fully vascularized bone equivalents. As with other avascular organoid systems, mild central hypoxia and diffusion limitations may occur in larger constructs, highlighting the need for future vascularization strategies, including endothelial co-culture, perfusable channels, organ-on-chip culture, or bioprinting-based vascularization, to improve long-term viability and maturation. Third, AB is a complex botanical system. Although HPLC fingerprinting was provided to document the extract profile and batch traceability, the specific bioactive constituents responsible for organoid enhancement remain unidentified. Further phytochemical characterization and bioassay-guided fractionation are needed to define the active components and establish standardized quality-control specifications. Moreover, ERK/MAPK mechanistic experiments were conducted in monolayer BMSCs rather than three-dimensional bone organoids. Thus, these findings support a role for ERK/MAPK signaling in AB-induced osteogenesis in BMSCs but do not directly establish its involvement in the organoid phenotype. Future studies should validate this pathway directly in three-dimensional organoid systems. Finally, the clinical cohort was retrospective and should be interpreted as supportive evidence for the osteoregenerative relevance of AB in human fracture healing, rather than as direct clinical proof of organoid implantation [38]. These clinical findings should be interpreted as hypothesis-generating rather than causal, because the retrospective design cannot fully account for unmeasured confounding. Several clinically important determinants of fracture healing, including detailed fracture classification and severity, open versus closed injury status, fixation characteristics, rehabilitation compliance, NSAID and other medication exposure, and baseline radiographic severity, were not uniformly available in the retrospective dataset. Residual confounding likely remains, and the observed associations may reflect selection effects or unrecorded differences in baseline severity rather than a true biological effect of AB. These limitations do not diminish the significance of the findings, but rather define the next steps for the field: testing in load-bearing defect models, integrating vascularized organoid strategies, and clarifying the active fractions and signaling pathways potentially contributing to the enhanced osteogenic phenotype of AB-treated organoids [39,40].

Taken together, the significance of this work lies not only in showing that AB enhances osteogenesis, but in demonstrating that a traditional osteoactive botanical factor can be repurposed as a developmental instruction for bone organoids. Relative to previous studies, the principal advances of the present work are threefold: AB is transformed from a conventional herbal extract into an organoid-compatible bioactive cue; the therapeutic unit is upgraded from isolated cells or soluble factors to an implantable living osteogenic microtissue; and the concept is supported by a multi-level experimental framework spanning cell culture, organoid maturation, animal defect repair, and human fracture-healing observation. In this sense, AB-bone organoids represent more than an incremental extension of AB pharmacology or bone organoid engineering; they define a promising direction for biologically instructed bone regeneration.

## 5 Conclusion

In conclusion, AB functionally upgrades bone organoids, enhances osteogenic maturation *in vitro*, improves repair of critical-sized bone defects *in vivo*, and is associated with improved fracture-healing outcomes in an exploratory clinical cohort receiving systemic oral AB. Rather than acting as a simple soluble stimulant, AB serves as an organoid-compatible osteoregenerative cue that enhances the osteogenic state of bone organoids across scales. Together, these findings support AB-bone organoids as a promising regenerative strategy for bone defect repair and provide a translational rationale for further clinical evaluation of AB as an osteoregenerative agent; however, the feasibility of AB-bone organoid implantation in patients remains to be established in dedicated clinical trials.

## CRediT authorship contribution statement

**Chenxing Lu:** Writing – original draft, Visualization, Validation, Methodology, Investigation, Formal analysis, Data curation, Conceptualization. **Xudong Qin:** Writing – original draft, Visualization, Methodology, Formal analysis, Data curation. **Zihao Lin:** Writing – original draft, Validation, Software, Methodology, Formal analysis, Data curation. **Song Han:** Methodology, Formal analysis, Data curation. **Peng Ding:** Software, Methodology, Investigation. **Li Xu:** Methodology, Formal analysis, Data curation. **Xu Zheng:** Software, Formal analysis. **Bin Hu:** Writing – review & editing, Visualization, Validation, Supervision, Resources, Project administration. **Fenghua Chen:** Writing – review & editing, Validation, Supervision, Project administration, Funding acquisition, Conceptualization. **Di Wu:** Writing – review & editing, Writing – original draft, Validation, Supervision, Resources, Project administration, Funding acquisition, Conceptualization.

## Declaration of competing interest

The authors declare that they have no known competing financial interests or personal relationships that could have appeared to influence the work reported in this paper.

## Data Availability

Data will be made available on request.
