# Supplementary material for: Achyranthes bidentata–functionalized bone organoids enhance bone defect repair
Source: Mater Today Bio. 2026 Sep 28;41:103716. doi: 10.1016/j.mtbio.2026.103716 (PMC13659489; doi:10.1016/j.mtbio.2026.103716)
Supplement: Supplementary file 1 [file mmc1.docx]

**Achyranthes bidentata–functionalized bone organoids enhance bone defect repair**

Chenxing Lu^a, 1^, Xudong Qin^b, 1^, Zihao Lin^c, 1^, Song Han^a^, Peng Ding^a^, Li Xu^a^, Xu Zheng^d^, Bin Hu^e, *^, Fenghua Chen^a, **^, Di Wu^f, g, ***^

^a^ Department of Orthopedic Surgery, Taizhou Hospital of Traditional Chinese Medicine, Jiangsu, China.

^b^ School of Pharmaceutical Sciences, Guangzhou Medical University, Guangzhou, China.

^c^ Department of Orthopaedic Surgery, The First Affiliated Hospital of Ningbo University, Ningbo, Zhejiang, China.

^d^ Department of Orthopedics, Renji Hospital, Shanghai Jiao Tong University School of Medicine, Shanghai, China.

^e^ Institute of Microsurgery on Extremities, Shanghai Sixth People's Hospital Affiliated to Shanghai Jiao Tong University School of Medicine, Shanghai, China.

^f^ Department of Orthopedic Surgery, Shanghai Sixth People's Hospital Affiliated to Shanghai Jiao Tong University School of Medicine, Shanghai, China.

^g^ Division of Engineering in Medicine, Department of Medicine, Brigham and Women’s Hospital, Harvard Medical School, Cambridge, MA, USA.

⁎Corresponding author. bin_hu22@163.com

⁎⁎Corresponding author. fenghuachen1979@163.com

⁎⁎⁎Corresponding author. Department of Orthopedic Surgery, Shanghai Sixth People's Hospital Affiliated to Shanghai Jiao Tong University School of Medicine, Shanghai, China. wudipumch@sjtu.edu.cn

^1^ These authors contributed equally to this study.

Figure S1. Characterization of Achyranthes bidentata (AB). (A) Representative constituent of AB; (B) HPLC fingerprint of the AB aqueous extract used in this study.

Figure S2. (A) Representative gross morphology of bone organoids and AB-bone organoids (day 7); (B) Quantification of organoid size. Data are presented as mean ± SD.

Figure S3. Representative low- and high-magnification H&E and Masson’s trichrome images of the defect area at 12 weeks. Dashed boxes indicate the regions shown at higher magnification.

Figure S4. Representative low- and high-magnification immunohistochemical staining of COL-I, OPN, and OCN expression within the regenerative area. Dashed boxes indicate the regions shown at higher magnification.

Figure S5. Representative radiographic healing course in an AB-treated patient. Sequential anteroposterior and lateral radiographs of a 50-year-old man with a left tibia fracture treated with open reduction and intramedullary nailing, followed by oral AB (25 g twice daily) beginning 1 month post-surgery for 6 weeks. Images demonstrate progressive callus formation at 1 and 3 months, complete cortical bridging by 6 months, and advanced remodeling with restoration of medullary continuity at 12 months.

Figure S6. Representative radiographic healing course in a control patient. Sequential anteroposterior and lateral radiographs of a 49-year-old man with a left tibia fracture treated with open reduction and intramedullary nailing and standard postoperative management without AB. Images show delayed callus maturation compared with AB-treated cases, with persistent fracture-line visibility at 3 months, partial cortical bridging at 6 months, and basic union achieved at 12 months with inferior remodeling relative to the AB group.

| Gene Symbol | GenBank Accession | Forward Primer (5′–3′) | Reverse Primer (5′–3′) | Product Size (bp) |
| --- | --- | --- | --- | --- |
| *Col1a1* | NM_053304.1 | GAGCGGAGAGTACTGGATCG | GCTTCTTTTCCTTGGGGTTC | 140 |
| *Runx2* | NM_001278484.1 | TAACGGTCTTCACAAATCCTC | GGCGGTCAGAGAACAAACTA | 115 |
| *Opn* | NM_012881.2 | GACGGCCGAGGTGATAGCTT | CATGGCTGGTCTTCCCGTTGC | 156 |
| *Ocn* | NM_013414.1 | CACAGGGAGGTGTGTGAG | TGTGCCGTCCATACTTTC | 186 |
| *Gapdh* | NM_017008.4 | GGCACAGTCAAGGCTGAGAATG | ATGGTGGTGAAGACGCCAGTA | 143 |

Table S1. qPCR primers for osteogenic genes in rat.
